# Prevalence and risk factors for *Taenia solium* cysticercosis in school-aged children: A school based study in western Sichuan, People’s Republic of China

**DOI:** 10.1371/journal.pntd.0006465

**Published:** 2018-05-08

**Authors:** John J. Openshaw, Alexis Medina, Stephen A. Felt, Tiaoying Li, Zhou Huan, Scott Rozelle, Stephen P. Luby

**Affiliations:** 1 Division of Infectious Diseases and Geographic Medicine, Department of Medicine, Stanford University, Stanford, CA, United States of America; 2 Freeman Spogli Institute, Stanford University, Stanford, CA, United States of America; 3 Department of Comparative Medicine, Stanford University School of Medicine, Stanford, CA, United States of America; 4 Institute of Parasitic Diseases, Sichuan Centers for Disease Control and Prevention, Sichuan Province, Chengdu, Sichuan, People’s Republic of China; 5 West China School of Public Health, Sichuan University, Chengdu, Sichuan, People’s Republic of China; Christian Medical College, UNITED STATES

## Abstract

**Background:**

*Taenia solium* cysticercosis affects millions of impoverished people worldwide and can cause neurocysticercosis, an infection of the central nervous system which is potentially fatal. Children may represent an especially vulnerable population to neurocysticercosis, due to the risk of cognitive impairment during formative school years. While previous epidemiologic studies have suggested high prevalence in rural China, the prevalence in children as well as risk factors and impact of disease in low-resource areas remain poorly characterized.

**Methodology/Principal findings:**

Utilizing school based sampling, we conducted a cross-sectional study, administering a questionnaire and collecting blood for *T*. *solium* cysticercosis antibodies in 2867 fifth and sixth grade students across 27 schools in west Sichuan. We used mixed-effects logistic regression models controlling for school-level clustering to study associations between risk factors and to characterize factors influencing the administration of deworming medication. Overall prevalence of cysticercosis antibodies was 6%, but prevalence was significantly higher in three schools which all had prevalences of 15% or higher. Students from households owning pigs (adjusted odds ratio [OR] 1.81, 95% CI 1.08–3.03), from households reporting feeding their pigs human feces (adjusted OR 1.49, 95% CI 1.03–2.16), and self-reporting worms in their feces (adjusted OR 1.85, 95% CI 1.18–2.91) were more likely to have cysticercosis IgG antibodies. Students attending high prevalence schools were more likely to come from households allowing pigs to freely forage for food (OR 2.26, 95% CI 1.72–2.98) and lacking a toilet (OR 1.84, 95% CI 1.38–2.46). Children who were boarding at school were less likely to have received treatment for gastrointestinal worms (adjusted OR 0.58, 95% CI 0.42–0.80).

**Conclusions/Significance:**

Our study indicates high prevalences of cysticercosis antibodies in young school aged children in rural China. While further studies to assess potential for school-based transmission are needed, school-based disease control may be an important intervention to ensure the health of vulnerable pediatric populations in *T*. *solium* endemic areas.

## Author summary

The zoonotic tapeworm, *Taenia solium*, affects millions of impoverished people worldwide and can cause neurocysticercosis (NCC), an infection of the central nervous system which is potentially fatal. Hypothetically, children may be a vulnerable population to infection as neurological problems and cognitive impairment caused by NCC during formative school years may lead to poor academic performance, contributing to drop-out rates and, eventually, propagating cycles of poverty. We carried out a school-based study of *T*. *solium* cysticerosis in primary school-aged children in rural western Sichuan. Our results indicate high levels of *T*. *solium* exposure in young school-aged children in rural China. While further studies to assess disease transmission within schools are needed, school-based disease control may be an important intervention to ensure the health of pediatric populations at risk for infection.

## Introduction

Infection with the zoonotic tapeworm *Taenia solium* affects millions of people living in poverty throughout Asia, Africa, and Latin America [1]. Considered a neglected tropical disease, infection is linked to inadequate sanitation and hygiene, presence of free roaming pigs, and poverty [2]. Infection in humans has two manifestations: intestinal taeniasis where humans serve as the definitive hosts for the adult tapeworm which inhabits the gastrointestinal tract, and cysticercosis, a tissue infection where humans are the accidental dead-end host for the cystic larvae (cysticercus).

Intestinal infestation with the adult tapeworm develops when humans consume improperly cooked pork containing cysticerci. The consumed cyst is released in the small intestines where the adult worm develops, attaches to the intestinal wall, and liberates thousands of eggs, which are shed, along with gravid proglottids, in human feces. *T*. *solium* eggs contaminate the environment and are consumed by pigs that ingest human feces directly or indirectly through contaminated agricultural products. Once consumed, the larval forms encyst in porcine muscle completing the cycle [3]. Human cysticercosis develops when *T*. *solium* eggs are consumed by humans through auto-infection, consumption of contaminated food or water, or close contact with a tapeworm carrier [4]. Upon ingestion of mature eggs, the hatched parasite migrates to tissues throughout the body including muscle, sub-cutaneous tissues, orbits, and the central nervous system (CNS) [5–7].

Neurocysticercosis (NCC) develops when *T*. *solium* larva establishes itself in the CNS and may lead to morbidity which can be fatal [5,8,9]. NCC causes a range of symptoms depending on number, stage of involution, volume, and location of the lesions [10], including seizures, chronic headaches, focal neurological deficits, psychiatric disturbances, and cognitive impairment [3,11–15].

The full prevalence of NCC is difficult to establish, it is estimated to be responsible for 29% of acquired epilepsy in endemic areas [16], and has been identified as a leading cause of death from foodborne disease resulting in 2.8 million disability-adjusted life years lost in 2010 [2]. *T*. *solium* has been reported throughout China, with hyperendemic foci mainly in southwest regions [17]. Overall, NCC may affect up to an estimated 7 million people in China [1]. However, the risk factors for infection and impact of disease in rural areas remain poorly characterized [17,18].

Children may represent an especially vulnerable population to NCC, with resulting neurological problems and cognitive impairment during formative school years possibly leading to poor academic performance, contributing to high drop-out rates and, eventually, propagating cycles of poverty. Schools may represent centers of transmission combining poor hygienic standards and close contact in a large and vulnerable population. Despite this hypothetical risk, the prevalence of cysticercosis within schools has not been well evaluated.

Here we report the prevalence of cysticercosis antibodies and associated risk factors in school-aged children using school-based sampling in western Sichuan, People’s Republic of China.

## Methods

### Ethics statement

The school principals, who are the children’s legal guardians while they are boarding at school, provided initial written consent for student participation, and each student provided verbal assent prior to participating. Consent and assent status were documented by field staff, and students who did not assent did not participate in the questionnaire or blood collection. Field staff and school staff described the purpose of the study using pre-written scripts and were available on hand to answer questions that students might have either about the study or questionnaires. Information on the study and a consent form were part of the take-home questionnaire, requesting written consent from adult caretakers in regard to their and their family member’s involvement. All participants were allowed to keep a copy of the consent/assent document. The study and all methodology were approved by the institutional review board of Stanford University (study ID 35415) and the ethical review board of West China School of Public Health, Sichuan University (K2015031).

### Sampling

The data used in this analysis were collected as part of an epidemiological field study conducted in November 2015 investigating *T*. *solium* cysticercosis and NCC prevalence, associations between NCC infection and academic performance, and risk factors for infection in school-aged children. Results of the cognitive and academic assessments, prevalences of NCC cases, and frequency of neurologic problems will be presented in another manuscript and are not further discussed here.

The study was carried out in rural mountainous areas of western Sichuan. Located at the eastern extremity of the Tibetan Plateau and with an average altitude of 2700 meters, these areas are largely characterized by smallholder farmers who raise pigs and partake in small-scale agriculture. These areas were selected as they had been identified as having high endemicity of *Taenia* species based on smaller scale studies [18]. In these remote areas, there is no routine mass antihelminthic drug administration for pediatric or adult populations.

To collect data which could be generalizable to school-aged children living in farming and pig raising communities in western Sichuan with known *T*. *solium* risk factors we used a school-based sampling technique. The study was conducted in three counties, each from one of three prefectures in western Sichuan. In selecting the three study counties (S1 Fig), we considered all counties within Aba (13 counties total), Ganzi (18 counties total), and Liangshan (16 counties and 1 autonomous county total) prefectures for the study. All counties in these prefectures are known to have smallholder pig raising activity and risk factors for human cysticercosis including open defecation and free range pigs based on reports from local public health workers to Sichuan Centers for Disease Control and Prevention (Sichuan CDC). Because we wanted to enroll students at risk for *T*. *solium* cysticercosis, we only considered counties where previous cases of human cysticercosis and NCC had previously been detected by Sichuan CDC or where there were previous reports to suggest *T*. *solium* taeniasis [18]. We further favored counties with the largest populations of fifth and sixth grade students as estimated by demographic data collected previously by colleagues at Sichuan CDC. Because these mountainous communities are often difficult to access due to poor roads and long distances, we focused on counties where all schools could be accessed by field teams and where local public health services were supportive of the field work.

All schools enrolling fifth and sixth graders were sampled within the three selected counties and all students in both grades were sampled at each school. We selected fifth and sixth grade students for the cognitive study because standardized tests can be administered to this age group easily and these students are old enough to have repeat and significant exposures.

At each school, field teams administered a student questionnaire, aseptically collected approximately 5 ml of blood by venous puncture for *T*. *solium* cysticercosis IgG testing by ELISA, and provided a take-home questionnaire to be completed by the child’s parent or guardian.

### Student and adult questionnaires

Student questionnaires (S2 Document**)** covered basic demographic data, home environment and family asset ownership, animal ownership, pork consumption, and student toileting behavior. If the family owned pigs, students were asked if they ever saw their family’s pigs eating human feces or if the pigs ever went to the areas where people defecated. While data on specific drugs could not reliably be collected, use of antihelminthics was assessed by asking students if they had taken any medication for “intestinal worms” in the past year. Finally, students were asked about symptoms and perceptions of intestinal worms. To assess if tapeworms or proglottids might be present in the child’s feces, students were asked if they had seen “worms” or “pieces of worms” in their feces.

Children were also provided with a take-home adult questionnaire which was completed by the head of household in their home. After completion, adult questionnaire forms were returned to the school by the child. On the adult questionnaire, head of households were asked about pig ownership and pig husbandry methods over the last year and slaughtering and meat preparation practices. Adults were also asked about agricultural practices in the five years preceding the questionnaire and about their knowledge and attitudes towards intestinal worms and the administration of medications for intestinal worms.

### Laboratory methods

Serum was tested using an enzyme-linked immunosorbent assay (ELISA) based on low-molecular-weight antigens (LMWAgs) of *T*. *solium* cysticerci collected from pigs in Chinese endemic areas. LMWAgs based assays have been shown to be highly sensitive and specific [19,20], have been used in previous field studies [21], and are especially attractive—given their low cost, quantifiable result, and simplicity—for use in low resource areas [20]. Detailed assay methodology has been published previously [20]. Antigen for the LMWAgs ELISA was obtained from cyst fluids of *T*. *solium* metacestodes collected from infected pigs in endemic areas of China. ELISA plates (Nunc-ImmunoTM plate, Maxisorp TM Surface, Thermo Fisher Scientific, Denmark) were coated with 100 μl of diluted LMWAgs at 1 μg/ml in PBS overnight at 4°C. Plates were rinsed twice with PBS containing 0.1% Tween 20 (PBST) and then blocked with 300 μl of blocking solution (10 mM Maleic acid pH 7.5, 150 mM NaCl, 1.0% casein, 0.1% Tween 20) at 37°C for 1 hour. Serum samples were diluted in blocking solution at 1:100. Plates with 100 μl of diluted sera in duplicate wells were incubated at 37°C for 1 hour. The wells were washed 3 times with PBST, incubated with 100μl of recombinant protein G conjugated with peroxidase (Invitrogen) at 1:4000 in blocking solution at 37°C for 1 hour. After washing 3 times with PBST, plates were incubated with 100 μl of substrate (0.4 mM 2,2’- azino-di-[3-ethyl-benzhiazoline sulfonate] in 0.1 M citric acid buffer, pH 5.3) for 30 minutes at 37°C. Color reaction was stopped by application of 1% SDS in each well. The optical density at 405 nm was evaluated with an ELISA reader. The cut-off point was determined as the mean optical density (OD) plus 3 times the standard deviation for a panel of serum samples obtained from healthy Chinese donors (n = 30).

### Statistical methods

We first characterized prevalence of cysticercosis antibodies, student demographics, reported toileting behaviors, and reported pig husbandry and agricultural practices using basic descriptive statistics. We constructed mixed-effects logistic regression models to better characterize differences in *T*. *solium* antibody seroprevalence at the school-level, to study associations between *T*. *solium* cysticercosis exposure and demographic, environmental, and behavioral factors, and to investigate what factors affected the likelihood of children receiving deworming medications in the year preceding the study.

We identified schools with higher student populations with *T*. *solium* cysticercosis antibodies by building mixed-effects logistic models with the serologic result as the dependent variable, school as the independent variable, and county as a random effect. A school with a cysticercosis antibody prevalence closest to the mean value in the dataset was used as the model reference.

**Fig 1:**
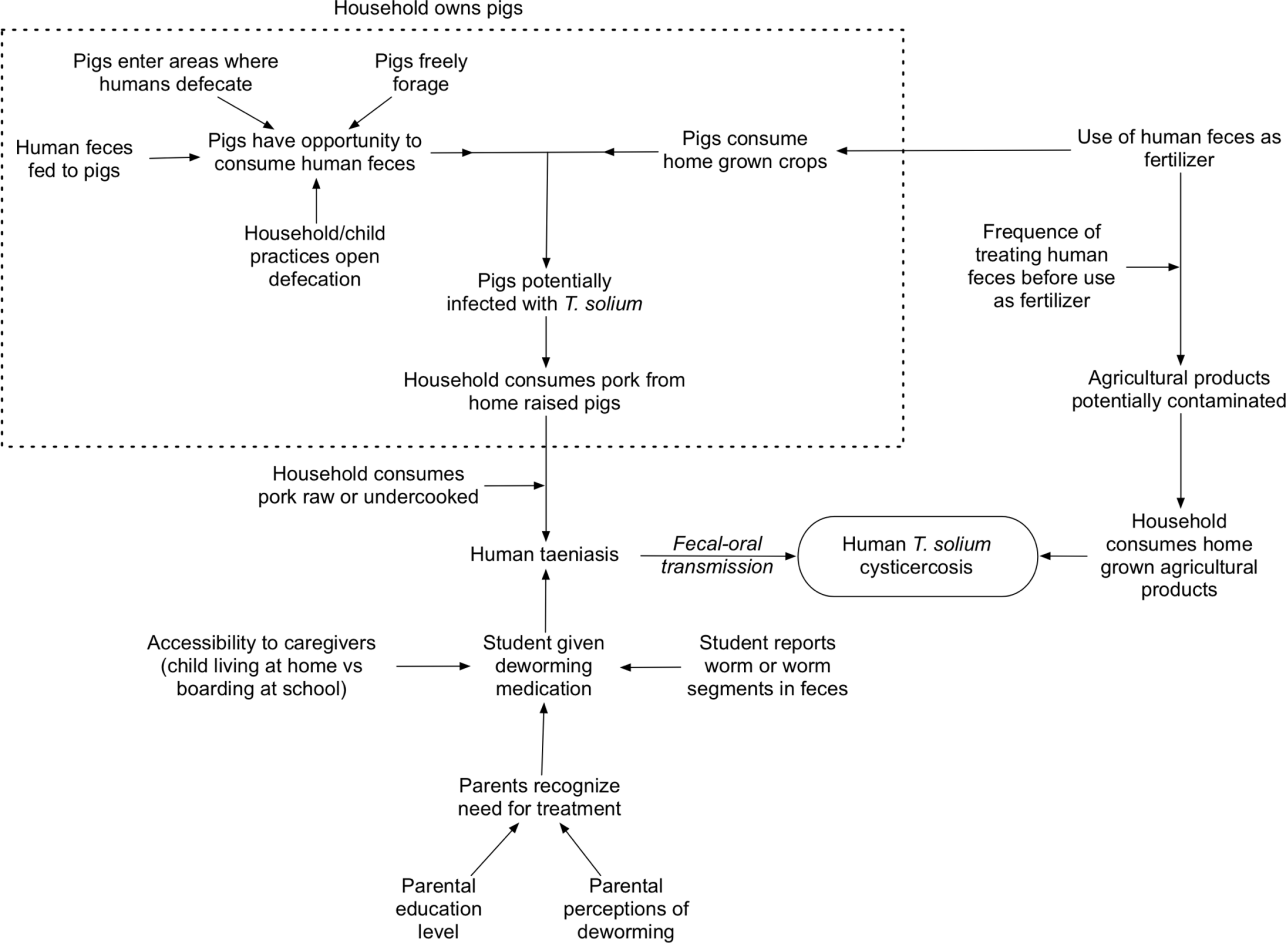
Diagram showing the relationship of variables included in the analysis to *T*. *solium* taeniasis and human cysticercosis infection.

To build models to assess the associations between risk factors and the presence of *T*. *solium* cysticercosis IgG antibodies or deworming medication administration, we first framed a causal diagram to identify associations between variables of interest (Fig 1) [22]. In the model assessing associations between risk factors and the presence of *T*. *solium* cysticercosis IgG antibodies we used the child’s serologic result for human cysticercosis IgG as the dependent variable. In the model assessing deworming medication administration we used the report of the child receiving medication for intestinal worms in the year preceding the study as the dependent variable.

Missing values in independent variables were imputed assuming an ignorable missingness mechanism using multivariate imputation by chained equations [23]. Numeric variables were imputed using predictive mean matching, logical variables using logistic regression, and categorical variables with more than two levels with multinomial logit models [23]. Predictors for multiple imputation included the independent variable in question, all dependent variables outlined in the causal diagram, demographics (age, sex, ethnicity, and asset score), and geographic location (school and county). Fifty imputed datasets were generated. Conditional rules were applied to ensure that imputation did not create impossible combinations (for example, a non-crop growing household reporting using human waste as crop fertilizer). Finally, results obtained using multiple imputation were compared both to available-case and complete-case analyses (shown in supplemental tables). All results reported within the manuscript are from multiple imputed analyses. Odds ratios were calculated by pooling results across all imputed datasets.

For each of the two dependent variables, we first constructed mixed-effects models consisting of a single independent variable controlling for school clustering as a random effect. To select variables to include in a best-fit multivariable mixed-effects logistic regression model, we used a two-step approach. Five imputed datasets were used for variable selection, and the final selected models were run on all fifty imputed datasets [24]. We first used information-theoretic model selection to generate all possible combinations of independent variables which had resulted in p-values less than 0.1 in our initial analysis and selected the model with the lowest corrected Akaike information criterion (AIC) for each of the five imputed dataset [25]. In the second variable selection step, variables selected in at least 50% of the five imputed datasets were assessed by backwards selection and retained if the Wald test resulted in a p value of less than 0.05 [24, 26]. To add a measure of wealth to our models we used principal components analysis (PCA) to aggregate asset ownership variables into one standardized asset score [27].

To identify if exposures and reported behaviors differed between students attending schools with the highest seroprevalences of *T*. *solium* IgG antibiodies compared to students attending the lowest prevalence schools, we compared the proportion of students reporting selected behaviors and exposures in the highest prevalence schools to students in all remaining lower prevalence schools using Fisher’s Exact Test.

Independent continuous variables included in mixed-effects models were confirmed to be linearly related to the log odds. We assessed multicollinearity between independent variables in multivariable models using variance inflation factors.

Analysis was conducted in R [28] utilizing the lme4 package [29] for logistic mixed-effects models, the MuMIn package for information-theoretic model selection [30], and the MICE package to perform multivariate imputation by chained equations [23].

## Results

A total of 3036 fifth and sixth grade students completed the student questionnaire. A total of 169 students refused to give blood, resulting in a final total of 2867 students with both serologic and exposure data included in the analysis. The highest refusal proportion was in Yajiang County where 8% (104/1242) of students refused to give blood, this was followed by Muli County where 5% refused (55/1071) and Ruoergai County where 2% (12/728) refused. The higher refusal proportion in Yajiang was driven by a single school were several teachers reportedly suggested their students refuse blood draws and 30% (35/118) of students in the targeted study population within the school refused to participate. Refusal proportions were 5% in the fifth-grade population (74/1634) and 7% (97/1407) in the sixth-grade population. The final study sample consisted of 1016 students in 12 schools in Muli County, 713 students in 9 schools in Ruoergai County, and 1138 students in 6 schools in Yajiang County. Of the 2867 students included in the analysis, 211 failed to return the take-home household questionnaire, resulting in a dataset including both student and parental reported data for 2656 students.

Enrolled students (Table 1) had a mean and median age of 13; 52% were female (1474/2847); 84% were of Tibetan ethnicity (2398/2866); and the majority boarded in school dormitories (65%, 1852/2861), sleeping and eating most meals during the week at school and returning to their village at regular intervals on weekends.

**Table 1:** Demographic and reported exposures and behavioral characteristics of study population.

Factor			Total (%)
**Demographics**	Age	≤ 10 years	31/2847 (1%)
		11–13 years	1915/2847 (67%)
		14–16 years	840/2847 (30%)
		17 or older	61/2847 (2%)
	Grade	5th grade	1560/2867 (54%)
	Sex	Male	1373/2847 (48%)
	Ethnicity	Tibetan	2398/2866 (84%)
		Han	95/2866 (3%)
		Miao	19/2866 (1%)
		Mongolian	21/2866 (1%)
		Yi	149/2866 (5%)
		Other	184/2866 (6%)
	Household asset score	1st Quartile (Poorest)	519/2524 (21%)
		2nd Quartile	752/2524 (30%)
		3rd Quartile	599/2524 (24%)
		4th Quartile (Wealthiest)	654/2524 (26%)
**Family Pig Ownership and Husbandry Practices**	Family currently owns pigs		2107/2843 (74%)
	Frequency pigs are allowed to freely forage in pig-owning households	Never	515/1735 (30%)
		Occasionally	733/1735 (42%)
		Always	487/1735 (28%)
	Pig-owning households reporting human feces in household fed to pigs		513/2425 (21%)
**Family Slaughtering Practices and Child Pork Consumption**	Age of pig at slaughter in pig-owning households	< 1 year	118/1694 (7%)
		1–2 years	575/1694 (34%)
		> 2 years	749/1694 (44%)
		Pigs never slaughtered	252/1694(15%)
	Pig-owning households reporting use of a commercial butcher to slaughter pigs		104/1705 (6%)
	Children reporting that consumed pork comes from family's home raised pigs		1737/2825 (61%)
	Head of households in pig-owning households that slaughter pigs reporting cysts in pork in last 5 years		304/1435 (21%)
	Frequency of pork consumption reported by children	Never	79/2865 (3%)
		1–2x/month	553/2865 (19%)
		3–5x/month	557/2865 (19%)
		6–10x/month	755/2865 (26%)
		≥11/month	921/2865 (32%)
	Children report consuming raw pork in last year		431/2781 (15%)
**Family Agricultural Practices**	Head of household reports growing crops		2202/2470 (89%)
	In crop growing households, head of household reports family consumes crops that they grow		2106/2184 (96%)
	In crop growing households, use of crops as pig fed	Crops fed to pigs	1493/2052 (73%)
		Crops not fed to pigs	150/2052 (7%)
		No pigs owned	409/2052 (20%)
	In crop growing households, head of household reports using human feces as a fertilizer		696/2101 (33%)
	If human feces are used as a fertilizer in crop growing households, frequency of treating prior to use	Never treat	217/687 (32%)
		Sometimes treat	358/687(52%)
		Always treat	112/687 (16%)
**Toilet and Child Toileting Behavior**	Family home has no toilet		1095/2861 (38%)
	Child reports defecating someplace other than bathroom		1327/2867 (46%)

Pig ownership was commonly reported, with 74% of households (2107/2843) owning pigs at home. Families reported owning a median of 3 pigs. Seventy percent of household heads (1220/1735) in pig-owning households reported that their family allowed their pigs to freely forage in the surrounding village and mountains. Sixty-one percent (1060/1724) of household heads in pig-owning households reported that they had observed their pigs consuming human feces in the environment while foraging, while 21% (513/2425) reported their pigs consumed human feces from their own household.

Pork consumption was almost universal, with only 3% of children reporting that they never consumed pork (79/2865). Sixty-one percent (1737/2825) of children reported that pork they consumed at home was raised in their household. Fifteen percent (431/2781) of children reported consuming undercooked pork in the past month. In the take-home household questionnaire, 21% (304/1435) of head of households in pig-owning households that slaughtered pigs reported seeing “cysts” in freshly butchered pork in the five years preceding the study.

Adults in the majority of households (89%, 2202/2470) reported growing crops. Crops were generally for household animal and human consumption and less commonly used as a source of cash income: 96% (2106/2184) consumed their own crops, 73% (1493/2052) fed their crops to their pigs, and 30% (635/2148) sold their crops for profit. Human feces were used as a fertilizer in 33% (696/2101) of households that grew crops. Human feces were often not treated—for example by composting or fermentation—before applying to crops. Of the households that reported using human feces as a fertilizer, 31% (217/687) reported never treating and only 16% (112/687) always treated prior to using.

Thirty-eight percent (1095/2861) of children reported having no toilet in their home. Forty-six percent (1330/2870) of children reported defecating someplace other than a toilet, with the two most common locations for outdoor defecation reported as within the village limit but outside the courtyard of their home (29%, 391/1330) and in the fields surrounding the village (55%, 740/1330).

### Prevalence of self-reported fecal worms and serum *T*. *solium* cysticercosis IgG antibodies

Overall, 11% (283/2606, 95% confidence interval [CI] 10–12%) of all fifth and sixth grade children self-reported that they had seen what appeared to be worms or worm segments in their feces (Table 2). The highest prevalence was in Muli County, where 14% (141/1013, 95% CI 12–16%) reported worms or worm segments in their feces.

**Table 2:** Prevalence of self-reported fecal worms and serum *T*. *solium* cysticercosis IgG antibodies by county and grade level.

		5th Graders		6th Graders		Both 5th and 6th Graders	
	County	Prevalence	95% CI	Prevalence	95% CI	Prevalence	95% CI
**Worms or worm segments in feces, self-reported in the year preceding study**	All	169/1420 (12%)	10–14%	114/1186 (10%)	8–11%	283/2606 (11%)	10–12%
	Muli	85/554 (15%)	12–19%	56/459 (12%)	9–16%	141/1013 (14%)	12–16%
	Yajiang	62/639 (10%)	8–12%	38/492 (8%)	6–11%	100/1131 (9%)	7–11%
	Ruoergai	22/227 (10%)	6–14%	20/235 (9%)	5–13%	42/462 (9%)	7–12%
**Serum Cysticercosis ELISA IgG Positive**	All	84/1560 (5%)	4–7%	96/1307 (7%)	6–9%	180/2867 (6%)	5–7%
	Muli	44/555 (8%)	6–11%	34/461 (7%)	5–10%	78/1016 (8%)	6–10%
	Yajiang	29/642 (5%)	3–7%	43/496 (9%)	6–12%	72/1138 (6%)	5–8%
	Ruoergai	11/363 (3%)	2–6%	19/350 (5%)	3–8%	30/713 (4%)	3–6%

Abbreviations: CI = confidence Interval

The overall prevalence of serum *T*. *solium* cysticercosis IgG antibodies in fifth and sixth grade students in the study area was 6% (180/2867, 95% CI 5–7%) (Table 2). The county prevalences in all enrolled fifth and sixth grade students ranged from 8% (78/1016, 95% CI 6–10%) in Muli County to 6% (72/1138, 95% CI 5–8%) in Yajiang County, and 4% (30/713, 95% CI 3–6%) in Ruoergai County.

**Fig 2:**
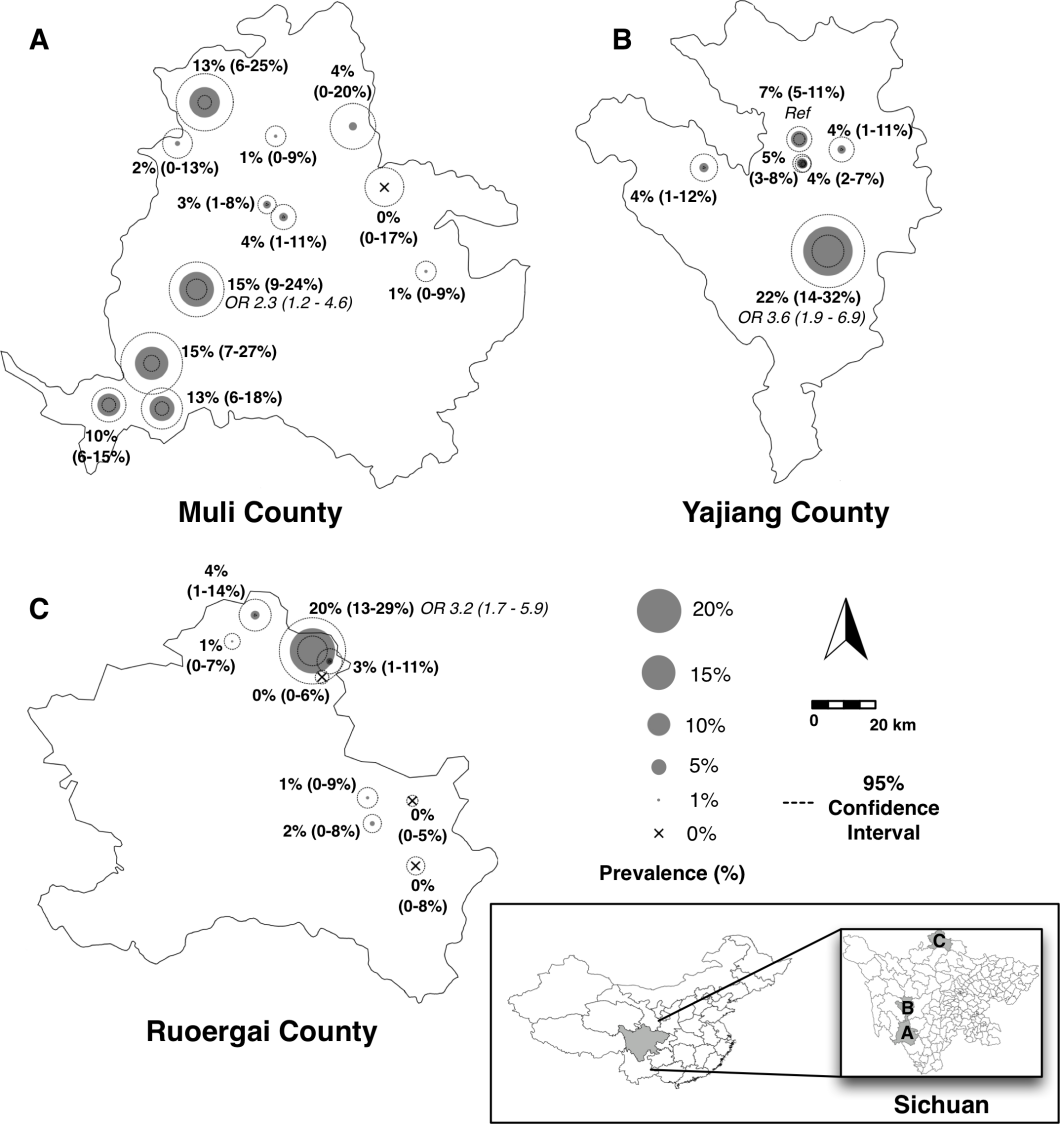
Prevalence of *T*. *solium* cysticercosis IgG antibiodies in 5^th^ and 6^th^ graders in three counties in Sichuan Province. *T*. *solium* cysticercosis antibody seropositivity in fifth and sixth graders in 27 schools across Muli (designated as A on map, 12 total schools), Yajiang (designated as B on map, 6 total schools), and Ruoergai (designated as C on map, 9 total schools) counties in western Sichuan. Gray shaded circles represent calculated prevalence and dotted circles represent 95% confidence intervals (CI). Bolded text shows corresponding prevalence and 95% CI. Odds ratios with 95% confidence intervals are displayed in italics for three schools that had a significantly higher prevalence compared to the school closest to the mean prevalence (noted as *Ref*). Inset shows location of the three study counties within Sichuan Province, People’s Republic of China.

Three schools had significantly higher prevalences of students with serum *T*. *solium* cysticercosis IgG antibodies when compared to a school with the mean prevalence in the study area (Fig 2): a school in Muli County with a prevalence of 15% (16/105, odds ratio [OR] 2.3, 95% CI 1.2–4.6), a school in Yajiang County with a prevalence of 22% (19/88, OR 3.6, 95% CI 1.9–6.9), and a school in Ruoergai County with a prevalence of 20% (22/111, OR 3.2, 95% CI 1.7–5.9). Four schools, three in Ruoergai County and one in Muli County, had no students with antibodies, although these results were not found to be statistically different than the mean prevalence in the study area.

### Risk factors for the presence of *T*. *solium* cysticercosis IgG antibodies

In mixed-effects logistic models consisting of a single independent variable (Table 3, see S1 Table for comparison of available-case, complete-case, and multiple imputed analyses) and controlling for school-level clustering, children were more likely to have IgG antibodies to *T*. *solium* cysticerci if they lived in households that owned pigs (4% vs 7%, OR 1.81, 95% CI 1.09–3.01) and if the family reported feeding household human feces to pigs (5% vs 11%, OR 1.54, 95% CI 1.07–2.24). While the odds ratio crossed one, suggesting no clear benefit, there was a trend suggesting treatment of human feces prior to use as a crop fertilizer resulted in less cysticercosis exposure: 6% of children had cysticerosis antibodies in households reporting never treating feces before use compared to 2% if human feces were always treated. There was also a trend towards more children with cysticercosis antibodies in households that did not own toilets (5% compared to 8%). Children who reported worms or worm segments in their feces were more likely to have serologic evidence of *T*. *solium* cysticercosis (10% compared to 6%, OR 1.60, 95% CI 1.03–2.5).

**Table 3:** Factors associated with presence of serum *T*. *solium* cysticercosis IgG antibodies.

Factor (N, % missing)			N (%) Seropositive	Single variable + School Clustering	Multivariate + School Clustering (best-fit model)
				Pooled* OR (95% CI)	Pooled* Adjusted OR (95% CI)
**Demographics**	Age (20 missing, <1%)	Continuous		1.10 (0.99–1.23)†	--
	Sex (20 missing, <1%)	Male	89 (6%)	Ref	--
		Female	88 (6%)	0.92 (0.68–1.25)	--
	Ethnicity (1 missing, <1%)	Tibetan	153 (6%)	Ref	--
		Han	6 (6%)	1.17 (0.46–2.95)	--
		Miao	1 (5%)	1.09 (0.13–9.09)	--
		Mongolian	2 (10%)	0.85 (0.18–4.01)	--
		Yi	2 (1%)	0.28 (0.06–1.21)†	--
		Other	16 (9%)	0.79 (0.35–1.75)	--
	Household asset score (343 missing, 12%)	1st Quartile (Poorest)	26 (5%)	Ref	--
		2nd Quartile	41 (5%)	1.07 (0.63–1.8)	--
		3rd Quartile	41 (7%)	1.18 (0.68–2.07)	--
		4th Quartile (Wealthiest)	47 (7%)	0.93 (0.51–1.72)	--
	Child boarding at school (6 missing, <1%)	No	56 (6%)	Ref	--
		Yes	123 (7%)	0.85 (0.55–1.29)	--
**Family Pig Ownership and Husbandry Practices**	Household owns pigs (24 missing, <1%)	No	28 (4%)	Ref	Ref
		Yes	150 (7%)	1.81 (1.09–3.01)**	1.81 (1.08–3.03)**
	Number of pigs owned (357 missing, 13%)	Continuous		1.02 (0.99–1.04)	--
	Frequency pigs allowed to forage (396 missing, 14%)	Never	31 (6%)	Ref	--
		Occasionally	50 (7%)	0.89 (0.55–1.44)	--
		Always	48 (10%)	1.13 (0.67–1.89)	--
	Household's human feces fed to pigs (442 missing, 15%)	No	105 (5%)	Ref	Ref
		Yes	54 (11%)	1.54 (1.07–2.24)**	1.49 (1.03–2.16)**
**Family Slaughtering Practices and Child Pork Consumption**	Household consumes home raised pigs (42 missing, 1%)	No	53 (5%)	Ref	--
		Yes	123 (7%)	1.23 (0.81–1.88)	--
	Frequency of pork consumption reported by children (2 missing, <1%)	Never	5 (6%)	Ref	--
		1–2x/month	29 (5%)	0.70 (0.26–1.88)	--
		3–5x/month	32 (6%)	0.74 (0.28–1.99)	--
		6–10x/month	45 (6%)	0.80 (0.30–2.1)	--
		≥11/month	68 (7%)	1.14 (0.43–3)	--
	Children report consuming raw pork in last year (86 missing, 3%)	No	142 (6%)	Ref	--
		Yes	32 (7%)	1.19 (0.79–1.78)	--
	Head of household noted cysts during butchering in last 5 years (444 missing, 16%)	No	81 (7%)	Ref	--
		Yes	24 (8%)	1.05 (0.64–1.71)	--
**Family Agricultural Practices**	Household grows crops (397 missing, 14%)	No	11 (4%)	Ref	--
		Yes	148 (7%)	1.33 (0.69–2.53)	--
	Pigs fed crops grown by household (364 missing, 13%)	No	9 (6%)	Ref	--
		Yes	111 (7%)	1.09 (0.53–2.26)	--
	Household reports use of human feces to fertilize crops (498 missing, 17%)	No	100 (7%)	Ref	--
		Yes	37 (5%)	0.90 (0.60–1.37)	--
	If human feces used as fertilizer, frequency of treating prior to use (507 missing, 18%)	Never treat	12 (6%)	Ref	--
		Sometimes treat	23 (6%)	0.85 (0.42–1.76)	--
		Always treat	2 (2%)	0.31 (0.07–1.36)	--
**Toilet and Child Toileting Behavior**	Family home has no toilet (6 missing, <1%)	No	93 (5%)	Ref	--
		Yes	87 (8%)	1.17 (0.83–1.66)	--
	Child reports defecating someplace other than bathroom (0 missing)	No	95 (6%)	Ref	--
		Yes	85 (6%)	0.97 (0.70–1.33)	--
**Worms or worm segments in feces and antihelminthic use**	Child self-reports worms or worm segments in feces in the last year (261 missing, 9%)	No	131 (6%)	Ref	Ref
		Yes	28 (10%)	1.6 (1.03–2.5)**	1.85 (1.18–2.91)**
	Child self-reports taking medication for gastrointestinal worms in last year (254 missing, 9%)	No	143 (6%)	Ref	Ref
		Yes	16 (4%)	0.60 (0.35–1.02)†	0.52 (0.31–0.90)**

Abbreviations: OR = odds ratio; CI = confidence interval; Ref = reference

*pooled analysis with 50 multiple imputed datasets

† = p < 0.1

** = p < 0.05; all factors resulting in p < 0.1 were considered in creation of best-fit multivariate model

The factors most associated with the presence of IgG antibodies to cysticercosis were pig ownership, feeding human feces to pigs, the presence of worms or worm segments in the child’s feces, and the child having been given medication for gastrointestinal worms (see S2 Table for variable selection results). In the multivariate model (Table 3), children who came from households that owned pigs and reported feeding the household’s human feces to their pigs were more likely to have serologic evidence of cysticercosis antibodies (adjusted OR 1.81, 95% CI 1.08–3.03 and adjusted OR 1.49, 95% CI 1.03–2.16, respectively). In the multivariate model, children who reported worms or worm segments in their feces were more likely to have antibodies (adjusted OR 1.85, 95% CI 1.18–2.91), children who reported receiving medication for gastrointestinal worms in the year preceding the study were less likely to have cysticercosis antibodies (adjusted OR 0.52, 95% CI 0.31–0.90).

### Comparison of demographics and risk factors in high and low *T*. *solium* cysticercosis antibody prevalence schools

**Fig 3:**
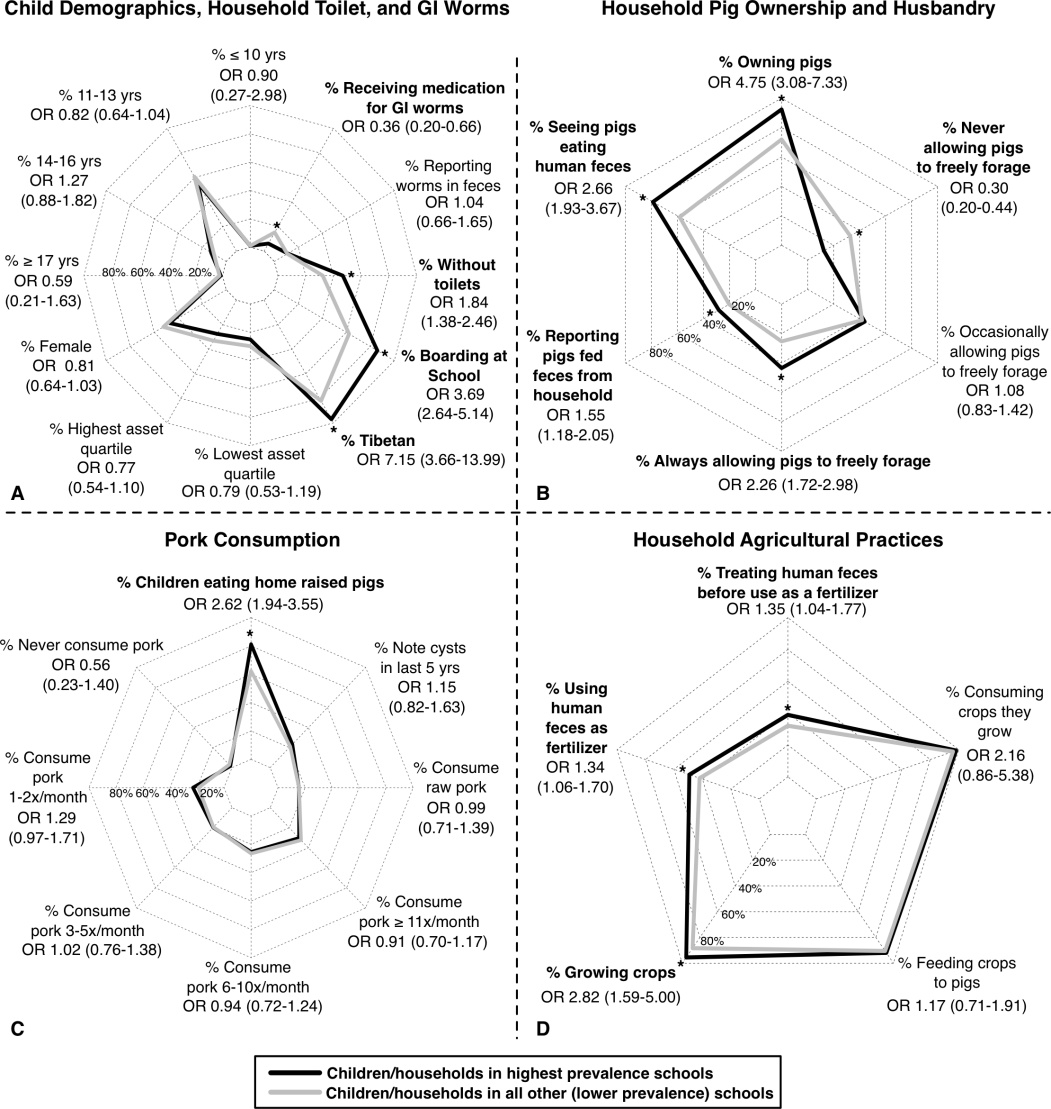
Comparison of demographic and behavioral factors between schools with highest and lower *T*. *solium* cysticercosis antibody prevalences. Proportion of demographic, household environment, and deworming (A); pig ownership and husbandry (B); pork consumption (C); and agricultural (D) factors reported by children or corresponding head of household in the three schools with the highest seroprevalences of *T*. *solium* IgG antibiodies (in black) compared to all other lower prevalence schools (in gray). Factors achieving statistical significance by Fisher’s exact test are bolded and the odds ratio (OR) and 95% confidence interval (CI) shown. * = p-value of < 0.05.

Students attending the three schools with the highest seroprevalences of *T*. *solium* IgG antibiodies differed from those attending schools with lower prevalences in reported demographics, behaviors, and exposures (Fig 3). Students attending the highest prevalence schools were more likely to be Tibetan (OR 7.15, 95% CI 3.66–13.99), report boarding at school (OR 3.69, 95% CI 2.64–5.14), and come from households without toilets (OR 1.84, 95% CI 1.38–2.46). Students attending the highest prevalence schools were less likely to have received medication for gastrointestinal worms in the year preceding the study (OR 0.36, 95% CI 0.20–0.66) (Fig 3A).

Students attending the highest prevalence schools were more likely to come from pig owning households (OR 4.75, 95% CI 3.08–7.33), report that their household’s human feces were fed to pigs (OR 1.55, 95% CI 1.18–2.05), and report that they had seen pigs consuming human feces in the environment (OR 2.66, 95% CI 1.93–3.67) (Fig 3B). Students attending the highest prevalence schools were more likely to come from households reporting always allowing their pigs to freely forage (OR 2.26, 95% CI 1.72–2.98) and less likely to come from households not allowing their pigs to forage (OR 0.30, 95% CI 0.20–0.44). While the frequency of reported pork consumption (Fig 3C) was the same in both student populations, students attending the highest prevalence schools were more likely to report that their pork came from home raised pigs (OR 2.62, 95% CI 1.94–3.55).

Differences in agricultural practices were less pronounced (Fig 3D), although children attending the highest prevalence schools were slightly more likely to come from households growing crops (OR 2.82, 95% CI 1.59–5.00), using human feces as a fertilizer (OR 1.34, 95% CI 1.06–1.70), and attempting to treat human feces through fermentation or composting before use as a fertilizer (OR 1.35, 95% CI 1.04–1.77).

### Factors influencing medication administration for gastrointestinal worms to school-aged children

Thirty-two percent of children reported having “intestinal worms” in the year preceding the study (818/2579). Most children reported that they had realized they were infected due to abdominal pain (55%, 448/818), while a smaller number reported seeing worms or worm pieces in their feces (20%, 163/817) or had been told that they had intestinal worms by a doctor (14%, 114/818).

When asked about their impression of gastrointestinal worms in their children, 35% (866/2469) of adult household respondents felt that intestinal worms had no adverse effects, 30% felt that worms could stunt children’s growth (765/2469), and 3% (69/2468) felt that worms could have a positive effect on children. When asked to identify the best treatments for gastrointestinal worms, 75% of adults (1832/2431) identified medication provided by a doctor, 10% (232/2431) suggested a combination of reducing outdoor activity and drinking hot water, and 9% (210/2431) suggested that spicy food should be consumed.

Fourteen percent (379/2613) of children reported that they had taken a medication for gastrointestinal worms in the year preceding the study. In mixed-effects logistic models consisting of a single independent variable (Table 4, see S3 Table for comparison of available-case, complete-case, and multiple imputed analyses) and controlling for school-level clustering, children who reported their ethnicity as “other” (OR 2.43, 95% CI 1.36–4.32) and who reported seeing worms or worm segments in their feces in the preceding year (OR 4.41, 95% CI 3.29–5.91) were more likely to have received treatment. Children who were older (OR 0.89, 95% CI 0.82–0.97), were of the Yi ethnicity (OR 0.48, 95% CI 0.23–0.99), were boarding at school (OR 0.52, 95% CI 0.38–0.70), and were wealthier as classified by household asset score (wealthiest quartile: OR 0.60, 95% CI 0.40–0.91) were less likely to have received medication for gastrointestinal worms in the year preceding the study.

**Table 4:** Factors associated with administration of medication for gastrointestinal worms to children.

Factor (N, % missing)			N (%) reporting medication for gastrointestinal worms in last year	Single variable + School Clustering	Multivariate + School Clustering (best-fit model)
				Pooled* OR (95% CI)	Pooled* Adjusted OR (95% CI)
**Demographics**	Sex (16 missing, <1%)	Male	178 (14%)	Ref	--
		Female	197 (15%)	0.99 (0.79–1.23)	--
	Age (18 missing, <1%)	Continuous		0.89 (0.82–0.97)**	0.91 (0.83–0.99)**
	Ethnicity (1 missing, <1%)	Tibetan	291 (14%)	Ref	--
		Han	15 (16%)	1.25 (0.67–2.34)	--
		Miao	2 (11%)	0.79 (0.17–3.71)	--
		Mongolian	4 (19%)	1.42 (0.43–4.7)	--
		Yi	11 (7%)	0.48 (0.23–0.99)**	--
		Other	56 (30%)	2.43 (1.36–4.32)**	--
	Household asset score (107 missing, 4%)	1st Quartile (Poorest)	100 (19%)	Ref	--
		2nd Quartile	98 (13%)	0.74 (0.53–1.03)†	--
		3rd Quartile	63 (11%)	0.52 (0.35–0.77)**	--
		4th Quartile (Wealthiest)	108 (17%)	0.60 (0.40–0.91)**	--
	Child boarding at school (6 missing, <1%)	No	194 (20%)	Ref	Ref
		Yes	185 (11%)	0.52 (0.38–0.70)**	0.58 (0.42–0.80)**
**Parental Education and Beliefs**	Highest level of education achieved by most educated parent (45 missing, 2%)	No formal education	123 (14%)	Ref	Ref
		Did not finish primary school	83 (13%)	0.94 (0.69–1.28)	0.88 (0.64–1.21)
		Primary school	70 (14%)	1.33 (0.96–1.86)†	1.28 (0.90–1.80)
		Junior high school	44 (18%)	1.60 (1.08–2.38)**	1.48 (0.98–2.22)†
		High school or higher	34 (26%)	2.37 (1.48–3.79)**	1.81 (1.11–2.98)**
		Unknown	24 (16%)	1.34 (0.82–2.2)	1.22 (0.73–2.04)
	Parents believe GI worms cause no adverse effects (516 missing, 20%)	No	207 (15%)	Ref	Ref
		Yes	82 (11%)	0.72 (0.54–0.95)**	0.68 (0.51–0.92)**
	Parents willing to take deworming medication (534 missing, 20%)	No	23 (10%)	Ref	--
		Yes	207 (15%)	1.38 (0.88–2.19)	--
		Don't know	55 (11%)	1.01 (0.61–1.69)	--
**Evidence of gastrointestinal worms**	Child reports worms or worm segments in feces in last year (15 missing, <1%)	No	277 (12%)	Ref	Ref
		Yes	102 (36%)	4.41 (3.29–5.91)**	4.43 (3.29–5.98)**

Abbreviations: OR = odds ratio; CI = confidence interval; Ref = reference

*Pooled analysis with 50 multiple imputed datasets

† = p < 0.1

** = p < 0.05; all factors resulting in p < 0.1 were considered in creation of best-fit multivariate model

Parental educational level and impressions of gastrointestinal worm influenced if children received treatment. In families where adults reported worms having no adverse effects, fewer children received medication (OR 0.72, 95% CI 0.54–0.95). Higher levels of education were associated with increasing medication administration, with parents achieving a junior high education or higher more likely to provide medication (junior high school: OR 1.60, 95% CI 1.08–2.38; high school or higher: OR 2.37, 95% CI 1.48–3.79).

The factors most associated with children receiving medication for gastrointestinal worms were age, school boarding status, level of parental education, parental understanding of adverse events caused by gastrointestinal worms, and the child reporting worms or worm segments in their feces in the last year (see S4 Table for variable selection results). In the multivariate model (Table 4), children of more highly educated parents (high school or higher: adjusted OR 1.81, 95% CI 1.11–2.98) and with worms or worm segments in their feces (adjusted OR 4.43, 95% CI 3.29–5.98) were more likely to receive treatment for gastrointestinal worms. Children who were older (adjusted OR 0.91, 95% CI 0.83–0.99), boarding at school (adjusted OR 0.58, 95% CI 0.42–0.80), and who had parents who felt intestinal worm infestation had no adverse effects (adjusted OR 0.68, 95% CI 0.51–0.92) were less likely to receive medication.

## Discussion

Our study demonstrates high prevalence of *T*. *solium* cysticercosis antibodies in school-aged children in poor, pig-raising areas in western Sichuan. The use of schools as a unit, rather than the typical village, is a unique approach and reveals variation in *T*. *solium* antibody seroprevalence across schools in close geographic proximity.

We identified three schools with significantly higher prevalences of *T*. *solium* cysticercosis antibodies than surrounding schools. Schools with the highest prevalence of *T*. *solium* cysticercosis antibodies had differences in reported behaviors and exposures compared to lower prevalence schools, with higher proportions of students in the highest prevalence schools reporting the consumption of home raised pigs, living in households without toilets, and coming from households were the family’s pigs are allowed to freely forage and fed human feces.

The seroprevalence of cysticercosis in children varies widely in the literature, from approximately 20% of 10–19 years olds found to be antigen positive in a village based study in the Democratic Republic of Congo [31], to approximately 12% of 11–20 year olds having antibodies in a hyperendemic area of Peru [32], and a study in three provinces of Burkina Faso showing *T*. *solium* antigen prevalence ranging from 2.3% to 0.7% in the youngest cohorts [33]. Although it is difficult to compare results given differing laboratory methodology and our school centered approach, the prevalence of *T*. *solium* cysticercosis antibodies in the highest prevalence schools in our study seem similar to levels reported in children in high endemic areas.

The risk factors most associated with cysticercosis antibodies in children identified in our best-fit multivariate analysis included pig ownership, the child self-identifying worms or worm segments in their feces, and households allowing their pigs to consume human feces. The link between the presence of antibodies and a recent history of young children possibly passing proglottids is consistent with previously published literature [32]. It is unclear from our study how many children are tapeworm carriers and are auto-infecting themselves, although given the poor handwashing practices among young children [34], there is likely substantial risk for auto-infection. Another potential explanation for this finding is that children who are passing proglottids are, along with their family members, consuming undercooked pork and therefore are likely surrounded by multiple members of their household who are harboring intestinal tapeworms.

The consumption of human feces by pigs results in infected pork and likely results in higher proportions of human intestinal infestation with the adult *T*. *solium* tapeworm. Children and adults in the study area often report either pigs eating the household’s human feces or report seeing their pigs eating human feces while foraging in the environment. Free range pigs’ access to human feces—made easier by lack of latrines and open defecation—has been frequently identified as a risk factor for cysticercosis [33, 35–37]. The use and acceptance of human feces as pig feed has been recognized as affecting household practices and preferences, for example respondents in a qualitative study on latrine use in Zambia voiced concern that building latrines would result in less available pig feed [38].

Risk factors identified in the school comparison are also consistent with factors previously identified in the literature. Pig ownership has been identified as a risk factor for cysticercosis and taeniasis in studies in Africa and South America [33,39], and higher risk of seropositivity has been seen in households that consume home raised pigs [39].

While not always meeting criteria for statistical significance, our analysis did suggest trends between agricultural techniques and cysticercosis exposure. Our comparison of the highest with lower prevalence schools suggested that children who attend the highest prevalence schools are more likely to come from households that grow their own crops. The use of human feces as fertilizer for crops has been recognized as a potential risk factor in previous studies [40,41]. While some households reported treating human feces prior to use as fertilizer, this practice is not common and the effectiveness of household techniques is unclear, especially given that consistently achieving the required levels of temperature, pH, and dryness to deactivate *T*. *solium* eggs may be difficult in household latrines [42]. The role of treating human feces prior to use as fertilizer and other agricultural practices in reducing infection risk deserves further characterization and study.

Unlike previous published studies, our study did not show any association between serologic status and pork consumption levels [33,39]. Our failure to detect this is perhaps due to the high pork consumption in the area and the fact that children, who do not prepare their own meals at home or at school, are likely poor judges of their pork intake. Additionally, we did not find an association between serologic status and poverty level [33]. This may be related to economic status being similar across the entire study area or to little variation in risk factors with increasing wealth given the agricultural and remote nature of our study communities.

Given that cyticercosis cases cluster around tapeworm carriers [32, 35, 43], it is possible that schools are acting as centers for transmission in pediatric populations. Schools represent large congregations of children, and risk for fecal-oral transmission and passage of eggs from tapeworm carriers is likely high. If this is the case, efforts to reduce school fecal-oral transmission may serve as a tool to interrupt disease transmission.

Treating human tapeworm carriers with antihelminthic medication eliminates the adult tapeworm, destroying the source of infection and preventing human and porcine cysticercosis [44]. While we did not collect data on specific antihelminthic use, our study, which did include questions regarding administration of medication to treat gastrointestinal worms, suggests that few children receive therapy. More concerning, our analysis showed that children who are boarding at school were less likely to receive medication than students living at home. Treatment is most likely to be administered by more educated parents who are aware of the potential adverse effects of tapeworm infestation. If tapeworm carriers are present in schools, the distribution of antihelminthic medications at schools could decrease possible school-based transmission between students.

Our study has some weaknesses that limit our scientific inference. We used a LMWAgs ELISA to detect antibodies to human *T*. *solium* cysticercosis. While historically less sensitive and specific than enzyme-linked immunotransfer blot (EITB) assays [45], ELISA performance for serodiagnosis of human cysticercosis has improved with the development of more sophisticated methods for producing antigenic proteins [46]. Use of LMWAgs rather than crude cyst fluid results in improved performance and less cross-reactivity with other pathogens [20]. However, evaluation of LMWAgs based ELISAs continues to suggest some weak cross-reaction with alveolar and cystic echinococcosis [20]. Because echinococcosis is endemic throughout regions of northwestern Sichuan [47], cross-reactivity may be causing us to over-estimate the prevalence of human *T*. *solium* cysticercosis antibodies. In this case, we suspect that misclassification caused by cross-reactivity is minimal given that echinococcosis in northwestern Sichuan is more common in pastoral herding communities than the farming communities which inhabit the regions included in this work [47].

Because the overall larger study was designed to assess the relationship between NCC and cognitive outcomes, we selected counties and schools to maximize disease based in small initial studies suggesting presence of NCC and human cysticercosis in the study areas. This means that our results may not be fully representative over a larger geographical area, as prevalences of disease may be higher in our area of study.

Some of the risk factors that failed to achieve significance in the best-fit model are risk factors for gastrointestinal taeniasis and likely failed to achieve significance because our selected laboratory outcome was a serologic test for cysticercosis antibodies. Our measure of taeniasis was based on students self-reporting worms or worm segments in their feces. We were not able to confirm if these reported gastrointestinal infestations were caused by *T*. *solium* nor were we able to conduct large scale stool testing to detect cases of taeniasis. *T*. *saginata* and *T*. *asiatica* are known to be present in the region [18], so some of these self-reported cases may represent other *Taenia* species or soil transmitted helminths. Our findings do suggest that students reporting worms or worm segments in their feces are more likely to have *T*. *solium* antibodies. Given the likely inclusion of gastrointestinal worms other than *T*. *solium* in our data collection, we may be underestimating the risk for cysticercosis associated with *T*. *solium* taeniasis. Stool testing and laboratory confirmation will be required to better characterize the prevalence of taeniasis and better clarify the associated risk for cysticercosis in the study area.

Because we do not have infection data on pigs raised in the study area, we cannot correlate prevalence with presence and density of infected pigs. Because of the mountainous terrain, long distances, and presumed limited movement of both villagers and pig populations, it is very possible that prevalence of *T*. *solium* cysticerci in pigs may vary widely in areas that are geographically proximal but isolated due to terrain features, and this may explain some of the variation in human seroprevalence. Given the complex biology of *T*. *solium*, the addition of measures for gastrointestinal taeniasis in humans and prevalence of cysticercosis in pigs would provide a more complete picture of the disease ecology.

Finally, because our study is questionnaire based, children failing to answer questions and adults failing to return take home questionnaires may have limited our ability to make school specific characterizations. In this case, however, overall participation in the study across the entire geographic area was high.

We have shown a high prevalence of *T*. *solium* cysticercosis antibodies in school-aged children with school-based clustering. These findings raise concerns for NCC in school-aged children and possible cognitive deficits caused by CNS infection, which could result in long-term negative health, economic, and social effects. *T*. *solium* is an eradicable disease. Combined approaches addressing community education, improvements in hygiene and sanitation, improved pig management and meat handling, treatment of tapeworm carriers with antihelminthics, and porcine treatment through vaccination and chemotherapy have shown success in reducing transmission [48–50]. While further work identifying tapeworm carriers and potential routes of transmission within schools is needed, our work raises the hypothesis that schools may be sites of *T*. *solium* cysticercosis transmission and that school based interventions may, therefore, be an important addition to reduce disease among vulnerable pediatric populations in *T*. *solium* endemic areas.

## Supporting information

S1 Fig County selection. The number of counties that did not meet criteria and were removed from consideration at each step are shown. Final selection was made to maximize the number of children available and to ensure that work was logistically possible and local public health departments were supportive of the work.
(PDF)

S1 Table Factors associated with presence of serum *T*. *solium* cysticercosis IgG antibodies, showing results from available case analysis, complete case analysis, and multiple imputation analysis. (PDF)

S2 Table Variable selection for best-fit model, presence of serum *T*. *solium* cysticercosis IgG antibodies. (PDF)

S3 Table Factors associated with administration of medication for gastrointestinal worms to children, showing results from available case analysis, complete case analysis, and multiple imputation analysis. (PDF)

S4 Table Variable selection for best-fit model, factors associated with administration of medication for gastrointestinal worms to children. (PDF)

S1 Document STROBE statement. (DOC)

S2 Document Selected questions used in analysis. (DOCX)
