# Supplementary material for: Prevalence and risk factors for Taenia solium cysticercosis in school-aged children: A school based study in western Sichuan, People’s Republic of China
Source: PLoS Negl Trop Dis. 2018 May 8;12(5):e0006465. doi: 10.1371/journal.pntd.0006465 (PMC5959190; doi:10.1371/journal.pntd.0006465)
Supplement: S1 Fig — The number of counties that did not meet criteria and were removed from consideration at each step are shown. Final selection was made to maximize the number of children available and to ensure that work was logistically possible and local public health departments were supportive of the work. (PDF) [file pntd.0006465.s001.pdf]

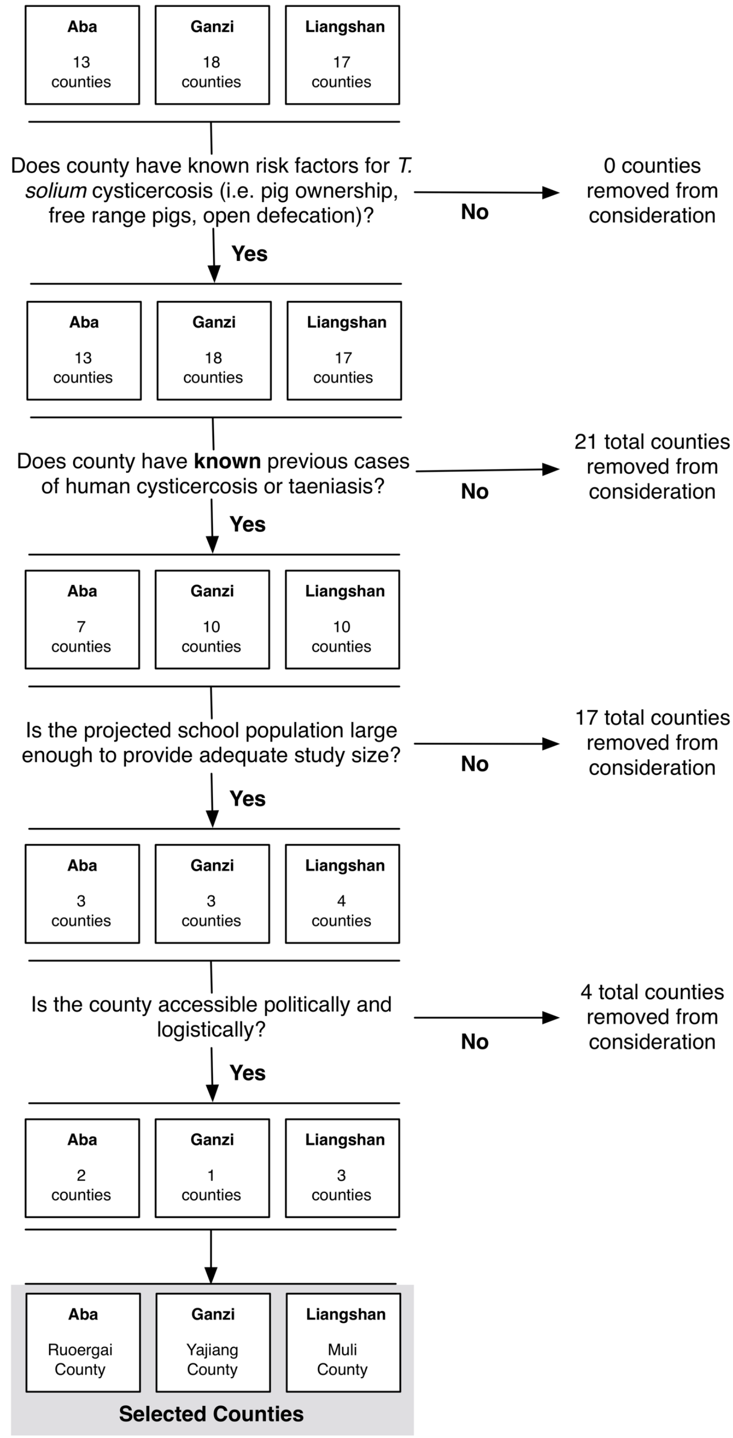

Supplemental Figure S1: County Selection

The number of counties that did not meet criteria and were removed from consideration at each step are shown. Final selection was made to maximize the number of children available and to ensure that work was logistically possible and local public health departments were supportive of the work.
