# Supplementary material for: Prevalence and risk factors for Taenia solium cysticercosis in school-aged children: A school based study in western Sichuan, People’s Republic of China
Source: PLoS Negl Trop Dis. 2018 May 8;12(5):e0006465. doi: 10.1371/journal.pntd.0006465 (PMC5959190; doi:10.1371/journal.pntd.0006465)
Supplement: S1 Table — (PDF) [file pntd.0006465.s002.pdf]

Supplemental Table S1: Factors associated with presence of serum *T. solium* cysticercosis IgG antibodies

		Available Case Analysis (n variable)			Complete Case Analysis (n = 1616)			Multiple Imputation Analysis (n = 2867; 50 iterations)	
Factor (N, % missing)		N (%) Seropositive	p value	OR	N (%) Seropositive	p value	OR	Pooled p value	Pooled OR
Age (20 missing, <1%)	Continuous		0.0795	1.1 (0.99 - 1.23)		0.4837	1.05 (0.91 - 1.21)	0.0854	1.1 (0.99 - 1.23)
Sex (20 missing, <1%)	Male	89 (6%)	Ref	Ref	50 (7%)	Ref	Ref	Ref	Ref
	Female	88 (6%)	0.6238	0.93 (0.68 - 1.27)	53 (6%)	0.7992	0.95 (0.63 - 1.43)	0.6	0.92 (0.68 - 1.25)
Ethnicity (1 missing, <1%)	Tibetan	153 (6%)	Ref	Ref	85 (7%)	Ref	Ref	Ref	Ref
	Han	6 (6%)	0.7457	1.17 (0.41 - 2.83)	3 (4%)	0.4673	0.63 (0.14 - 1.99)	0.7456	1.17 (0.46 - 2.95)
	Miao	1 (5%)	0.9379	1.09 (0.06 - 6.9)	1 (7%)	0.7547	1.41 (0.07 - 9.5)	0.9378	1.09 (0.13 - 9.09)
	Mongolian	2 (10%)	0.8385	0.85 (0.13 - 3.41)	1 (6%)	0.6698	0.63 (0.03 - 3.85)	0.8385	0.85 (0.18 - 4.01)
	Yi	2 (1%)	0.0872	0.28 (0.04 - 1.04)	2 (2%)	0.1707	0.35 (0.05 - 1.39)	0.0874	0.28 (0.06 - 1.21)
	Other	16 (9%)	0.5561	0.79 (0.34 - 1.75)	11 (10%)	0.6343	0.79 (0.29 - 2.02)	0.5563	0.79 (0.35 - 1.75)
Household asset score (343 missing, 12%)	1st Quartile (Poorest)	26 (5%)	Ref	Ref	19 (6%)	Ref	Ref	Ref	Ref
	2nd Quartile	41 (5%)	0.8746	1.04 (0.61 - 1.81)	25 (5%)	0.5867	0.84 (0.43 - 1.63)	0.8095	1.07 (0.63 - 1.8)
	3rd Quartile	41 (7%)	0.6140	1.16 (0.66 - 2.06)	31 (8%)	0.6004	1.19 (0.61 - 2.37)	0.5545	1.18 (0.68 - 2.07)
	4th Quartile (Wealthiest)	47 (7%)	0.8260	0.93 (0.5 - 1.76)	28 (7%)	0.6393	0.83 (0.38 - 1.82)	0.827	0.93 (0.51 - 1.72)
Child boarding at school (6 missing, <1%)	No	56 (6%)	Ref	Ref	40 (6%)	Ref	Ref	Ref	Ref
	Yes	123 (7%)	0.4257	0.84 (0.55 - 1.31)	63 (6%)	0.1833	0.71 (0.42 - 1.2)	0.44	0.85 (0.55 - 1.29)
Household owns pigs (24 missing, <1%)	No	28 (4%)	Ref	Ref	15 (4%)	Ref	Ref	Ref	Ref
	Yes	150 (7%)	0.0204	1.82 (1.1 - 3.09)	88 (7%)	0.0640	1.87 (0.97 - 3.75)	0.0221	1.81 (1.09 - 3.01)
Number of pigs owned (357 missing, 13%)	Continuous		0.2388	1.02 (0.99 - 1.04)		0.0588	1.03 (1 - 1.06)	0.2419	1.02 (0.99 - 1.04)
Frequency pigs allowed to forage (396 missing, 14%)	Never	31 (6%)	Ref	Ref	19 (6%)	Ref	Ref	Ref	Ref
	Occasionallly	50 (7%)	0.5875	0.87 (0.54 - 1.44)	29 (6%)	0.8634	0.95 (0.51 - 1.78)	0.6362	0.89 (0.55 - 1.44)
	Always	48 (10%)	0.5561	1.16 (0.7 - 1.95)	40 (11%)	0.1110	1.62 (0.89 - 3.03)	0.6441	1.13 (0.67 - 1.89)
Household's human feces fed to pigs (442 missing, 15%)	No	105 (5%)	Ref	Ref	69 (5%)	Ref	Ref	Ref	Ref
	Yes	54 (11%)	0.0180	1.57 (1.07 - 2.28)	34 (10%)	0.0472	1.6 (1.0 - 2.53)	0.0212	1.54 (1.07 - 2.24)

Household consumes home raised pigs (42 missing, 1%)	No	53 (5%)	Ref		22 (4%)	Ref	Ref	Ref	Ref
	Yes	123 (7%)	0.3280	1.23 (0.81 - 1.91)	81 (7%)	0.0651	1.71 (0.97 - 3.12)	0.3307	1.23 (0.81 - 1.88)
Frequency of pork consumption reported by children (2 missing, <1%)	Never	5 (6%)	Ref	Ref				Ref	Ref
	1-2x/month	29 (5%)	0.4714	0.69 (0.27 - 2.14)	19 (6%)	Ref	Ref	0.4734	0.7 (0.26 - 1.88)
	3-5x/month	32 (6%)	0.5596	0.75 (0.3 - 2.28)	17 (5%)	0.4880	0.79 (0.39 - 1.57)	0.5561	0.74 (0.28 - 1.99)
	6-10x/month	45 (6%)	0.6484	0.8 (0.32 - 2.41)	25 (5%)	0.9236	0.97 (0.51 - 1.86)	0.648	0.8 (0.3 - 2.1)
	≥11/month	68 (7%)	0.7919	1.14 (0.46 - 3.44)	42 (8%)	0.3113	1.38 (0.74 - 2.65)	0.7886	1.14 (0.43 - 3)
Children report consuming raw pork in last year (86 missing, 3%)	No	142 (6%)	Ref	Ref	84 (6%)	Ref	Ref	Ref	Ref
	Yes	32 (7%)	0.4308	1.18 (0.77 - 1.76)	19 (8%)	0.4580	1.22 (0.7 - 2.05)	0.4062	1.19 (0.79 - 1.78)
Head of household noted cysts during butchering in last 5 years (444 missing, 16%)	No	81 (7%)	Ref	Ref	57 (7%)	Ref	Ref	Ref	Ref
	Yes	24 (8%)	0.8400	1.05 (0.63 - 1.7)	18 (9%)	0.2642	1.38 (0.76 - 2.42)	0.8437	1.05 (0.64 - 1.71)
Household grows crops (397 missing, 14%)	No	11 (4%)	Ref	Ref	8 (5%)	Ref	Ref	Ref	Ref
	Yes	148 (7%)	0.3578	1.35 (0.73 - 2.75)	95 (7%)	0.9121	1.04 (0.5 - 2.46)	0.3907	1.33 (0.69 - 2.53)
Pigs fed crops grown by household (364 missing, 13%)	No	9 (6%)	Ref	Ref	4 (4%)	Ref	Ref	Ref	Ref
	Yes	111 (7%)	0.8427	1.08 (0.54 - 2.39)	82 (8%)	0.4349	1.51 (0.59 - 5.11)	0.8166	1.09 (0.53 - 2.26)
Household reports use of human feces to fertilize crops (498 missing, 17%)	No	100 (7%)	Ref	Ref	74 (7%)	Ref	Ref	Ref	Ref
	Yes	37 (5%)	0.6339	0.9 (0.59 - 1.37)	21 (5%)	0.4724	0.82 (0.47 - 1.39)	0.6353	0.9 (0.6 - 1.37)
If human feces used as fertilizer, frequency of treating prior to use (507 missing, 18%)	Never treat	12 (6%)	Ref	Ref	6 (4%)	Ref	Ref	Ref	Ref
	Sometimes treat	23 (6%)	0.6520	0.84 (0.40 - 1.84)	15 (6%)	0.7160	1.19 (0.46 - 3.51)	0.6734549	0.85 (0.42 - 1.76)
	Always treat	2 (2%)	0.1270	0.31 (0.04 - 1.22)				0.1203382	0.31 (0.07 - 1.36)
Family home has no toilet (6 missing, <1%)	No	93 (5%)	Ref	Ref	53 (5%)	Ref	Ref	Ref	Ref
	Yes	87 (8%)	0.3660	1.17 (0.82 - 1.67)	50 (8%)	0.5967	1.13 (0.71 - 1.79)	0.3653	1.17 (0.83 - 1.66)
Child reports defecating someplace other than bathroom (0 missing)	No	95 (6%)	Ref	Ref	56 (7%)	Ref	Ref	Ref	Ref
	Yes	85 (6%)	0.8322	0.97 (0.7 - 1.33)	47 (6%)	0.3804	0.83 (0.54 - 1.27)	0.8322	0.97 (0.7 - 1.33)
Child self-reports worms or worm segments in feces in the last year (261 missing, 9%)	No	131 (6%)	Ref	Ref	82 (6%)	Ref	Ref	Ref	Ref
	Yes	28 (10%)	0.0316	1.63 (1.02 - 2.51)	21 (12%)	0.0045	2.15 (1.23 - 3.62)	0.0379	1.6 (1.03 - 2.5)
	No	143 (6%)	Ref	Ref	93 (7%)	Ref	Ref	Ref	Ref

Child self-reports taking medication for gastrointestinal worms in last year (254 missing, 9%)	Yes	16 (4%)	0.0540	0.59 (0.33 - 0.99)	10 (4%)	0.1677	0.62 (0.3 - 1.18)	0.0585	0.6 (0.35 - 1.02)

Key

(pink)	p < 0.1
(green)	P < 0.05
(grey)	Factor level dropped due to lack of available data in complete case analysis

Available case and multiple imputation analysis yielded very similar results. Many cases had to be dropped in complete case analysis given missing data. Because of this, this analysis has much less power than other analyses. Despite this, overall trends are consistent across all three analyses.
