# Supplementary material for: Prevalence and risk factors for Taenia solium cysticercosis in school-aged children: A school based study in western Sichuan, People’s Republic of China
Source: PLoS Negl Trop Dis. 2018 May 8;12(5):e0006465. doi: 10.1371/journal.pntd.0006465 (PMC5959190; doi:10.1371/journal.pntd.0006465)
Supplement: S2 Table — (PDF) [file pntd.0006465.s003.pdf]

Supplemental Table S2: Variable selection, presence of serum *T. solium* cysticercosis IgG antibodies

Factor	p-values < 0.1 (answer, p-value)	Number of times variable included in best fit model by information-theoretic model selection (out of 5 imputed datasets)	Wald p	Inclusion in Model?
Age	Continuous variable; p = 0.0854	4/5	p = 0.09	Not included
Sex	None	NA	NA	Not included
Ethnicity	Yi, p = 0.0874	0/5	NA	Not included
Household asset score	None	NA	NA	Not included
Child boarding at school	None	NA	NA	Not included
**Household owns pigs**	**Yes, p = 0.0221**	**5/5**	**p < 0.05**	**Included**
Number of pigs owned	None	NA	NA	Not included
Frequency pigs allowed to forage	None	NA	NA	Not included
**Household's human feces fed to pigs**	**Yes, p = 0.0212**	**5/5**	**p < 0.05**	**Included**
Household consumes home raised pigs	None	NA	NA	Not included
Frequency of pork consumption reported	None	NA	NA	Not included
Children report consuming raw pork in last year	None	NA	NA	Not included
Head of household noted cysts during butchering in last 5 years	None	NA	NA	Not included
Household grows crops	None	NA	NA	Not included
Pigs fed crops grown by household	None	NA	NA	Not included
Household reports use of human feces to fertilize crops	None	NA	NA	Not included
If human feces used as fertilizer, frequency of treating prior to use	None	NA	NA	Not included
Family home has no toilet	None	NA	NA	Not included
Child reports defecating someplace other than bathroom	None	NA	NA	Not included
**Child self-reports worms or worm segments in feces in the last year**	**Yes, p = 0.0379**	**5/5**	**p < 0.05**	**Included**
**Child self-reports taking medication for gastrointestinal worms in last year**	**Yes, p = 0.0585**	**5/5**	**p < 0.05**	**Included**
