# Supplementary material for: Prevalence and risk factors for Taenia solium cysticercosis in school-aged children: A school based study in western Sichuan, People’s Republic of China
Source: PLoS Negl Trop Dis. 2018 May 8;12(5):e0006465. doi: 10.1371/journal.pntd.0006465 (PMC5959190; doi:10.1371/journal.pntd.0006465)
Supplement: S3 Table — (PDF) [file pntd.0006465.s004.pdf]

Supplemental Table S3: Factors associated with administration of medication for gastrointestinal worms to children

Factor (N, % missing)			Available Case Analysis (n variable)			Complete Cases (n = 1934)			Multiple Imputation Analysis (n = 2613; 50 iterations)	
			N (%) reporting medication for gastrointestinal worms in last year	p Value	OR (95% CI)	N (%) reporting medication for gastrointestinal worms in last year	p Value	OR (95% CI)	Pooled p value	Pooled OR
Demographics	Sex (16 missing, <1%)	Male	178 (14%)	Ref	Ref	136 (15%)	Ref	Ref	Ref	Ref
		Female	197 (15%)	0.9356	0.99 (0.79 - 1.24)	135 (13%)	0.2339	0.85 (0.66 - 1.11)	0.9093	0.99 (0.79 - 1.23)
	Age (18 missing, <1%)	Continuous		0.0067	0.89 (0.82 - 0.97)		0.0955	0.92 (0.84 - 1.01)	0.0066	0.89 (0.82 - 0.97)
	Ethnicity (1 missing, <1%)	Tibetan	291 (14%)	Ref	Ref	204 (13%)	Ref	Ref	Ref	Ref
		Han	15 (16%)	0.4767	1.25 (0.65 - 2.29)	14 (19%)	0.1118	1.71 (0.85 - 3.25)	0.4766	1.25 (0.67 - 2.34)
		Miao	2 (11%)	0.7666	0.79 (0.12 - 3.13)	2 (12%)	0.9737	1.03 (0.15 - 4.12)	0.7667	0.79 (0.17 - 3.71)
		Mongolian	4 (19%)	0.5624	1.42 (0.38 - 4.38)	4 (20%)	0.5009	1.51 (0.4 - 4.67)	0.5623	1.42 (0.43 - 4.7)
		Yi	11 (7%)	0.0478	0.48 (0.22 - 0.97)	10 (8%)	0.1683	0.59 (0.26 - 1.21)	0.0479	0.48 (0.23 - 0.99)
		Other	56 (30%)	0.0025	2.43 (1.36 - 4.34)	37 (29%)	0.0057	2.42 (1.28 - 4.5)	0.0026	2.43 (1.36 - 4.32)
	Household asset score (107 missing, 4%)	1st Quartile (Poorest)	100 (19%)	Ref	Ref	77 (20%)	Ref	Ref	Ref	Ref
		2nd Quartile	98 (13%)	0.0705	0.74 (0.53 - 1.03)†	73 (12%)	0.0170	0.63 (0.43 - 0.92)	0.0738	0.74 (0.53 - 1.03)
		3rd Quartile	63 (11%)	0.0010	0.52 (0.35 - 0.77)	46 (10%)	0.0009	0.47 (0.3 - 0.74)	0.0011	0.52 (0.35 - 0.77)
		4th Quartile (Wealthiest)	108 (17%)	0.0166	0.6 (0.4 - 0.91)	75 (16%)	0.0146	0.54 (0.33 - 0.88)	0.0165	0.6 (0.4 - 0.91)
	Child boarding at school (6 missing, <1%)	No	194 (20%)	Ref	Ref	151 (20%)	Ref	Ref	Ref	Ref
		Yes	185 (11%)	<0.0001	0.52 (0.38 - 0.71)	120 (10%)	<0.0001	0.46 (0.32 - 0.65)	<0.0001	0.52 (0.38 - 0.7)
Parental Education and Beliefs	Highest level of education achieved by most educated parent (45 missing, 2%)	No formal education	123 (14%)	Ref	Ref	74 (11%)	Ref	Ref	Ref	Ref
		Did not finish primary school	83 (13%)	0.7024	0.94 (0.69 - 1.28)	67 (14%)	0.2650	1.23 (0.85 - 1.76)	0.6878	0.94 (0.69 - 1.28)
		Primary school	70 (14%)	0.0834	1.34 (0.96 - 1.87)	46 (12%)	0.1708	1.33 (0.88 - 2)	0.0884	1.33 (0.96 - 1.86)
		Junior high school	44 (18%)	0.0199	1.6 (1.07 - 2.37)	36 (18%)	0.0061	1.88 (1.19 - 2.95)	0.0195	1.6 (1.08 - 2.38)
		High school or higher	34 (26%)	0.0003	2.38 (1.47 - 3.79)	30 (29%)	0.0000	3.5 (2.05 - 5.91)	0.0003	2.37 (1.48 - 3.79)
		Unknown	24 (16%)	0.2507	1.33 (0.8 - 2.16)	18 (15%)	0.1440	1.53 (0.84 - 2.69)	0.2382	1.34 (0.82 - 2.2)

	Parents believe GI worms cause no adverse effects (516 missing, 20%)	No	207 (15%)	Ref	Ref	199 (16%)	Ref	Ref	Ref	Ref
		Yes	82 (11%)	0.0182	0.71 (0.53 - 0.94)	72 (11%)	0.0060	0.66 (0.49 - 0.89)	0.0216	0.72 (0.54 - 0.95)
	Parents willing to take deworming medication (534 missing, 20%)	No	23 (10%)	Ref	Ref	21 (10%)	Ref	Ref	Ref	Ref
		Yes	207 (15%)	0.1182	1.45 (0.92 - 2.38)	195 (15%)	0.0839	1.54 (0.96 - 2.57)	0.1638	1.38 (0.88 - 2.19)
		Don't know	55 (11%)	0.9561	1.01 (0.6 - 1.75)	55 (12%)	0.5848	1.16 (0.68 - 2.04)	0.9637	1.01 (0.61 - 1.69)
**Evidence of gastrointestinal worms**	Child reports worms or worm segments in feces in last year (15 missing, <1%)	No	277 (12%)	Ref	Ref	195 (11%)	Ref	Ref	Ref	Ref
		Yes	102 (36%)	<0.0001	4.41 (3.28 - 5.92)	76 (35%)	<0.0001	4.66 (3.31 - 6.54)	<0.0001	4.41 (3.29 - 5.91)

Key

Colour	Meaning
Pink	p < 0.1
Green	P < 0.05

Available case and multiple imputation analysis yielded very similar results. Many cases had to be dropped in complete case analysis given missing data. Because of this, this analysis has much less power than other analyses. Despite this, overall trends are consistent across all three analyses.
