# Supplementary material for: Prevalence and risk factors for Taenia solium cysticercosis in school-aged children: A school based study in western Sichuan, People’s Republic of China
Source: PLoS Negl Trop Dis. 2018 May 8;12(5):e0006465. doi: 10.1371/journal.pntd.0006465 (PMC5959190; doi:10.1371/journal.pntd.0006465)
Supplement: S4 Table — (PDF) [file pntd.0006465.s005.pdf]

Supplemental Table S4: Variable selection, Factors associated with administration of medication for gastrointestinal worms to children

Factor	p-values < 0.1 (answer, p-value)	Number of times variable included in best fit model by information-theoretic model selection (out of 5 imputed datasets)	Wald p	Inclusion in Model?
Sex	None	NA	NA	Not included
**Age**	**Continuous variable: p = 0.006**	**5/5**	**p < 0.05**	**Included**
Ethnicity	Yi, p = 0.0479<br>Other, p = 0.0026	0/5	NA	Not included
Household asset score	2nd Quartile, p = 0.0738<br>3rd Quartile, p = 0.0011<br>4th Quartile, p = 0.0165	0/5	NA	Not included
**Child boarding at school**	**Yes, p < 0.0001**	**5/5**	**p < 0.05**	**Included**
**Highest level of education achieved by most educated parent**	**Primary school, p = 0.0884**<br>**Junior high school, p = 0.0195**<br>**High school or higher, p = 0.0003**	**5/5**	**p < 0.05**	**Included**
**Parents believe GI worms cause no adverse effects**	**Yes, p = 0.0216**	**5/5**	**p < 0.05**	**Included**
Parents willing to take deworming medication	None	NA	NA	Not included
**Child reports worms or worm segments in feces in last year**	**Yes, p < 0.0001**	**5/5**	**p < 0.05**	**Included**
