# Supplementary material for: Prevalence and risk factors for Taenia solium cysticercosis in school-aged children: A school based study in western Sichuan, People’s Republic of China
Source: PLoS Negl Trop Dis. 2018 May 8;12(5):e0006465. doi: 10.1371/journal.pntd.0006465 (PMC5959190; doi:10.1371/journal.pntd.0006465)
Supplement: S1 Document — (DOC) [file pntd.0006465.s006.doc]

**Prevalence and risk factors for *Taenia solium* cysticercosis in school-aged children: a school based study in western Sichuan, People’s Republic of China**

**Supplemental S1 Document: STROBE Statement—checklist of items that should be included in reports of observational studies**

|  | Item No | Recommendation | Comments |
| --- | --- | --- | --- |
| **Title and abstract** | 1 | (*a*) Indicate the study’s design with a commonly used term in the title or the abstract | Included in abstract |
|  |  | (*b*) Provide in the abstract an informative and balanced summary of what was done and what was found | Done |
| Introduction | | |  |
| Background/rationale | 2 | Explain the scientific background and rationale for the investigation being reported | See first three paragraphs of introduction |
| Objectives | 3 | State specific objectives, including any prespecified hypotheses | See last three paragraphs of introduction |
| Methods | | |  |
| Study design | 4 | Present key elements of study design early in the paper | Done in methods section |
| Setting | 5 | Describe the setting, locations, and relevant dates, including periods of recruitment, exposure, follow-up, and data collection | See sampling section in methods |
| Participants | 6 | *Cross-sectional study*—Give the eligibility criteria, and the sources and methods of selection of participants | See sampling section in methods |
|  |  | (*b*) *Cohort study*—For matched studies, give matching criteria and number of exposed and unexposed  *Case-control study*—For matched studies, give matching criteria and the number of controls per case | N/A |
| Variables | 7 | Clearly define all outcomes, exposures, predictors, potential confounders, and effect modifiers. Give diagnostic criteria, if applicable | See methods sections describing questionnaires and laboratory |
| Data sources/ measurement | 8* | For each variable of interest, give sources of data and details of methods of assessment (measurement). Describe comparability of assessment methods if there is more than one group | See methods sections describing questionnaires and laboratory |
| Bias | 9 | Describe any efforts to address potential sources of bias | See statistical techniques section in methods; mixed-effects models controlled for school based clustering. |
| Study size | 10 | Explain how the study size was arrived at | See sampling section of methods |
| Quantitative variables | 11 | Explain how quantitative variables were handled in the analyses. If applicable, describe which groupings were chosen and why | Quantitative variables included were age and number of pigs owned. These were analysed as continuous variables. All other variables were categorical based on the multiple-choice questionnaire. |
| Statistical methods | 12 | (*a*) Describe all statistical methods, including those used to control for confounding | See statistical methods section |
|  |  | (*b*) Describe any methods used to examine subgroups and interactions | See statistical methods section |
|  |  | (*c*) Explain how missing data were addressed | See statistical methods section; multiple imputation was used as described in the methods |
|  |  | (*d*) *Cross-sectional study*—If applicable, describe analytical methods taking account of sampling strategy | See statistical methods section |
|  |  | (*e*) Describe any sensitivity analyses | See statistical methods section |

| Results | | |  |
| --- | --- | --- | --- |
| Participants | 13* | (a) Report numbers of individuals at each stage of study—eg numbers potentially eligible, examined for eligibility, confirmed eligible, included in the study, completing follow-up, and analysed | See 1^st^ paragraph in results |
|  |  | (b) Give reasons for non-participation at each stage | See 1^st^ paragraph in results |
|  |  | (c) Consider use of a flow diagram | Given this could easily be described, left as text |
| Descriptive data | 14* | (a) Give characteristics of study participants (eg demographic, clinical, social) and information on exposures and potential confounders | See Table 1 |
|  |  | (b) Indicate number of participants with missing data for each variable of interest | See Tables 3 and 4 for full details on amount of missing data |
|  |  | (c) *Cohort study*—Summarise follow-up time (eg, average and total amount) | NA |
| Outcome data | 15* | *Cohort study*—Report numbers of outcome events or summary measures over time | *NA* |
|  |  | *Case-control study—*Report numbers in each exposure category, or summary measures of exposure | *NA* |
|  |  | *Cross-sectional study—*Report numbers of outcome events or summary measures | See Tables 1, 2, 3 |
| Main results | 16 | (*a*) Give unadjusted estimates and, if applicable, confounder-adjusted estimates and their precision (eg, 95% confidence interval). Make clear which confounders were adjusted for and why they were included | Unadjusted and adjusted analysis are shown for all analyses |
|  |  | (*b*) Report category boundaries when continuous variables were categorized | Done |
|  |  | (*c*) If relevant, consider translating estimates of relative risk into absolute risk for a meaningful time period | NA |
| Other analyses | 17 | Report other analyses done—eg analyses of subgroups and interactions, and sensitivity analyses | All analyses as described in methods are presented |
| Discussion | | |  |
| Key results | 18 | Summarise key results with reference to study objectives | Done |
| Limitations | 19 | Discuss limitations of the study, taking into account sources of potential bias or imprecision. Discuss both direction and magnitude of any potential bias | Done, see discussion section on limitations |
| Interpretation | 20 | Give a cautious overall interpretation of results considering objectives, limitations, multiplicity of analyses, results from similar studies, and other relevant evidence | Done |
| Generalisability | 21 | Discuss the generalisability (external validity) of the study results | Done |
| Other information | | |  |
| Funding | 22 | Give the source of funding and the role of the funders for the present study and, if applicable, for the original study on which the present article is based | See financial disclosure |

*Give information separately for cases and controls in case-control studies and, if applicable, for exposed and unexposed groups in cohort and cross-sectional studies.

**Note:** An Explanation and Elaboration article discusses each checklist item and gives methodological background and published examples of transparent reporting. The STROBE checklist is best used in conjunction with this article (freely available on the Web sites of PLoS Medicine at http://www.plosmedicine.org/, Annals of Internal Medicine at http://www.annals.org/, and Epidemiology at http://www.epidem.com/). Information on the STROBE Initiative is available at www.strobe-statement.org.
