# Supplementary material for: Prevalence and risk factors for Taenia solium cysticercosis in school-aged children: A school based study in western Sichuan, People’s Republic of China
Source: PLoS Negl Trop Dis. 2018 May 8;12(5):e0006465. doi: 10.1371/journal.pntd.0006465 (PMC5959190; doi:10.1371/journal.pntd.0006465)
Supplement: S2 Document — (DOCX) [file pntd.0006465.s007.docx]

**Prevalence and risk factors for *Taenia solium* cysticercosis in school-aged children: a school based study in western Sichuan, People’s Republic of China**

**Supplemental S2 Document: Selected Questions Used in Analysis**

**Student Demographics and Household Wealth**

| **Survey** | **Text** | **Answer choices** |
| --- | --- | --- |
| Student | What is your birthdate using the Gregorian Calendar? | Free entry |
| Student | What year were you born? | Free entry |
| Student | What is your zodiac sign? | Horse, Sheep, Monkey, Chicken, Dog, Pig, Other |
| Student | What is your ethnicity? | Tibetan, Han, Qiang, Yi, Miao, Hui, Mongolian, Other |
| Student | Are you currently boarding at school this semester? | Yes, No |
| Student | Does your family have the following items?  Electric bike or motorcycle  Truck or car  Refrigerator  Air conditioner  Washing machine  Running water  Water heater  Flush toilet  Computer | Yes, No |
| Student | What is the highest level of education achieved by your mother / father? | Illiterate, unschooled; Did not graduate from primary school; Primary school; Junior/Middle School; High school (academic or vocational); University or above; Not sure/unclear |

**Family Pig Ownership and Husbandry Practices**

| **Survey** | **Text** | **Answer choices** |
| --- | --- | --- |
| Student / Adult | Does your family own pigs? | Yes; No |
| Student / Adult | How many pigs does your family own? | Free entry |
| Student | Do your family’s pigs ever go to the area where you defecate? | We do not own pigs; Never; Very rarely; Sometimes; Often; Very often |
| Student | Do your family’s pigs ever eat your stool after you defecate? | We do not own pigs; Never; Very rarely; Sometimes; Often; Very often |
| Adult | In the last year, did your pigs ever wander into areas where they can find and eat human waste? | Never; Rarely; Occasionally; Most days; Every day |
| Adult | Have you ever seen your pigs eating human waste? | Never; Rarely; Occasionally; Most days; Every day |
| Adult | In the last year, did you let your pigs loose to forage and find their own food? | Never; Rarely; Occasionally; Most days; Every day |
| Adult | In the last year, did you purposefully feed your pigs human waste? | Never; Rarely; Occasionally; Most days; Every day |
| Adult | In the last year, did you ever feed your pigs crops you grow yourself? | Never; Rarely; Occasionally; Most days; Every day |
| Adult | In the last five years, when you slaughtered your pigs, did you ever see cysts or white spots in their tissue or tongue? | Yes; No |
| Adult | Does your family slaughter the pigs that you own? | Yes; No |
| Adult | At what age do you generally slaughter your pigs for food? | 1 year or less; 1-2 years; 2 years or greater |

**Agricultural**

| **Survey** | **Text** | **Answer choices** |
| --- | --- | --- |
| Adult | In the last five years, did you grow crops? | Yes; No |
| Adult | Do you consume crops that you grow? | Never; Rarely; Occasionally; Most days; Every day |
| Adult | Do you use your crops to feed pigs? | Yes; No |
| Adult | Do you use human feces to fertilize your crops? | Yes; No |
| Adult | Do you ever decontaminate the human feces you are using to fertilize your crops, for example by composting or fermentation? | Never; Occasionally; Most of the time; Always |

**Pork Consumption**

| **Survey** | **Text** | **Answer choices** |
| --- | --- | --- |
| Student | In the last month, how often did you (adult questionnaire: your family) eat pork? | Never; Very rarely (Once or twice a month); Sometimes (3-5 times per month); Often (6-10 times per month); Very often (11 or more times per month) |
| Student/Adult | When you eat pork at home, where does your family usually get the meat? | It comes from our own pigs; We buy it from another family; We buy it from a store; Other; Don’t know |
| Student/Adult | In the last month, did you (adult questionnaire: your family) ever eat raw or undercooked pork? | Never; Very rarely (Once or twice a month); Sometimes (3-5 times per month); Often (6-10 times per month); Very often (11 or more times per month); Don’t know |
| Student/Adult | In the last year, have you ever noticed white spots or cysts in pork during slaughter, meal preparation or eating? | Never; Very rarely; Sometimes; Often; Very often |

**Toileting Habits**

| **Survey** | **Text** | **Answer choices** |
| --- | --- | --- |
| Student / Adult | What type of toilet does your family most frequently use at home? | None; Pit toilet (no cement); Cement pit toilet; Ceramic pot; Flush toilet; Other |
| Student / Adult | What happens to human waste in your toilet? (check all that apply) | It enters the sewage system; It is eaten by pigs; It is eaten by animals other than pigs; It is used to fertilize crops; Other |
| Student | Do you ever defecate anywhere other than the bathroom? | Never; Very rarely; Sometimes; Often; Very often |
| Student | If you defecate somewhere other than the bathroom, where specifically do you go? (Check all that apply) | I never do this; Inside the house; In the courtyard; Outside the courtyard, but around the house; In the field Other |

**Intestinal Worms and Antihelminthics**

| **Survey** | **Text** | **Answer choices** |
| --- | --- | --- |
| Student | In the past year, have you been infected with intestinal worms (have worms in your tummy)? | Yes; No |
| Student | How did you know that you had been infected by intestinal worms? : Saw worms when I defecated | Yes; No |
| Student | In the last year, how often did you see worms pieces in your stool? | Never; 1-2 times per year; Every 2-3 months; Monthly or more |
| Student | Within the past year, did you take any medicine for intestinal worms? | Yes; No |
| Adult | What kind of effect do you think infection with intestinal worms has on a child? | No effect; It will make the child feel thirsty; It will make the child feel hungry; It will retard the child’s growth; It has a positive effect |
| Adult | Which of the following do you think is the best way to treat an infection with intestinal worms? | Eat very spicy food; Get a doctor to prescribe deworming medicine; Reduce outdoor activity and drink plenty of hot water; There is no way to treat intestinal worm infections; Other |
| Adult | How do you view giving deworming medicine to your family? | Give preventative deworming medicine once a year; Give deworming medicine only after an examination has shown infection; There is no need to give medicine; I don’t know |
| Adult | If you were infected with intestinal worms, would you be willing to take deworming medicine? | Yes; No; I don’t know |
